# ACL Graft Selection Based on Age and Sport

**DOI:** 10.1177/26350254241308584

**Published:** 2025-03-11

**Authors:** Mert Kahraman Marasli, Berte Bøe

**Affiliations:** † Haydarpasa Numune Training and Research Hospital, Istanbul, Turkey; ‡ Department of Orthopaedic, Oslo University Hospital, Oslo, Norway; Investigation performed at Oslo University Hospital, Oslo, Norway

**Keywords:** ACL, graft choice, graft selection, return to sport, sideline management

## Abstract

**Background:**

The issue of graft selection in anterior cruciate ligament (ACL) reconstruction continues to be debated in the literature. It has been reported in the literature that different graft types give different results, especially in different demographic characteristics (age, sex) and different sports. In this study, we attempted to provide an overview of how graft selection should be made according to demographic characteristics (age, sex) and sport types.

**Indications:**

In this study, we tried to review the studies in the literature that compared graft types in ACL reconstruction according to age, sex, or type of sport.

**Technique Description:**

A narrow review of the literature was performed to compare graft selection specific to different sports.

**Results:**

There is no perfect graft selection for everyone. Given the distinct characteristics of each sport branch, including the grade of pivoting, frequency of repeated movements, and expectations, it is evident that dedicated research is required for each sport. Comparative studies on graft selection specific to each sport may increase the accuracy of the selection.

**Discussion/Conclusion:**

A review of the literature reveals a paucity of studies on the selection and outcomes of grafts in different sports. When choosing a graft for ACL reconstruction, factors such as age, sex, sport type, and expectations should be taken into consideration and a personalized decision should be made.

**Patient Consent Disclosure Statement:**

The author(s) attests that consent has been obtained from any patient(s) appearing in this publication. If the individual may be identifiable, the author(s) has included a statement of release or other written form of approval from the patient(s) with this submission for publication.

## Graphical abstract

**Graphical Abstract:**
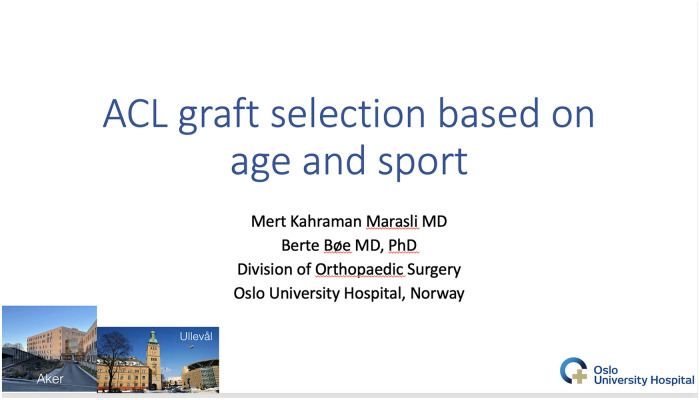
This is a visual representation of the abstract.

## Video Transcript

Our contribution to this *VJSM* special edition is on anterior cruciate ligament (ACL) graft choice based on age and sport. The first author is Mert Kahraman Marasli and the last author and presenter is Berte Bøe.

Dr Marasli has no disclosures and Dr Boe’s disclosures are paid lectures from Smith & Nephew and Ortomedic.

The outline of this presentation is to present a short background of the strength of the native ACL measured in biomechanical studies. We will present the graft options and how age, sex, and sport might influence our choice.

## Background

The native ACL maximum tensile strength measured by load to failure in young specimens has been estimated by several biomechanical studies and has been found to be approximately 2200 N, and the linear stiffness has been estimated to be approximately 242 N/mm. As age increases, the estimated load to failure and stiffness for the native ACL have been shown to decrease significantly. The estimated load to failure was 658 N, with a stiffness of 180 N/mm, for specimens aged between 60 and 97 years.^1^ It is of the utmost importance to ensure that the anatomic and biomechanical properties of an ACL graft are aligned with those of the native ACL to minimize the risk of ACL graft failure.

The ACL rupture represents more than 50% of all knee injuries and affects more than 200,000 people in the United States each year.^17^

The graft options for ACL reconstruction are either auto- or allo-grafts. For autografts, there are the patellar tendon with bone plugs (BTB), the hamstring tendon, or the quadriceps tendon (QT). Care must be taken when attempting to directly compare biomechanical models of the mechanical properties of grafts because differences in mechanisms by which grafts are placed under tension and tested can greatly affect the observed biomechanical values between trials.^1^

Is there any perfect graft choice for ACL reconstruction? In this editorial commentary, Freedman^10^ postulates that “there is no perfect graft choice for everyone. If there was, there wouldn’t be a choice!”

## Results

We know that the difference in terms of outcomes between grafts decreases with increasing age. The risk of graft rupture is very high when using allografts in patients younger than 25 years.^4^ In a systematic review, Wasserstein et al^23^ examined the outcomes of allograft versus autograft reconstruction of the ACL in patients younger than 25 years. The review reported a 9.6% graft failure rate with autograft compared to a 25.0% revision rate with allograft in this young, active patient population.

In a report from the Norwegian Cruciate Ligament Registry, 14,201 primary ACL reconstructions among alpine skiers, soccer players, and handball players were enrolled from 2004 to 2016. The graft survival rates were calculated for individuals in each of the 3 sport types, for BTB and hamstring tendon grafts separately, and related to age at primary operation. Graft revision rate was 1.8 times higher for hamstring than for BTB and 2.8 times higher for individuals 18 years old and younger. This supports the use of BTB grafts in this age group if the growth zones in the knee are closed.^5^

How about graft selection in older patients? In this study from Sha et al,^19^ 44 patients older than 45 years underwent primary ACL reconstruction. Neither the method used to create the femoral tunnel, nor the graft type used in ACL reconstruction, caused a significant difference in postoperative patient-reported outcome measures with a minimum 2-year follow-up in this group. Meena et al^16^ reported on 57 patients older than 50 years with primary arthroscopic ACL reconstruction using a quadraceps tendon autograft. The group of highly active older patients provided satisfactory patient-reported functional outcomes and allowed recovery to the preinjury level of activity. The authors conclude that the QT autograft is a good graft option in patients older than 50 years, but there was no control group comparing data with other grafts in this study.

Let’s move to graft selection according to sex. Etzel et al^8^ published on a cohort of females younger than 25 years. They included 1385 female patients and compared 655 BTB versus 525 hamstring tendon autografts. There was a significant difference in mean failure rate between BTB (6.1%) and hamstring grafts (17.4%). In conclusion, female patients 25 years and younger showed significantly lower graft failure with BTB than hamstring tendon.

We have some criteria to consider the optimal graft selection for athletes engaged in different sports:

- Return-to-sport rates (RTS)
- Graft rupture rates (revision rates)
- Time taken to return to sport (TTRS)
- Athletic performance on return to sport^15^

For athletes playing soccer:

- There were no differences between BTB and hamstring tendon autografts in RTS. This conclusion is based on data from the Swedish and Italian professional first leagues, on both sexes, and on data from the MOON group.^2,22^
- In a study on collegiate female soccer players, authorized by Howard et al,^13^ there were no differences between graft types in RTS.

Walden et al^22^ looked into the TTRS in soccer.

- They found no differences between BTB and hamstring tendon autografts in time to return to training or match play.

For American football:

- There was no statistically significant difference (*P* = .3206) when comparing return-to-play (RTP) rates among players who received a BTB autograft versus those who received a hamstring tendon autograft.
- There was a significant difference in RTP rates when comparing players who received an autograft of any kind (with 85% RTP) versus those who received an allograft (with 69% RTP).^3^

In 2 different cohorts of rugby players, Hurley et al^14^ published on a cohort of 126 players in whom the BTB graft was used in all cases. They found a graft rupture rate of 1.8%. In the other cohort from Takazawa,^21^ all rugby players were treated with a hamstring autograft. In this cohort of 121 rugby players, they reported a 16% ACL graft rupture. This indicates that the hamstring autograft may not be an appropriate graft source to use in a younger active population, including rugby players.

Harris et al^12^ have published a report on basketball players:

- Unfortunately, the numbers are too small to conclude on differences between grafts.

We have some data from skiers and snowboarding as well. The RTS is overall high, but we have no information about graft types and revision rates in these reports.^7,11^

For ice hockey players, we would like to present 2 studies. Erickson et al^6^ published on 36 National Hockey League (NHL) players.

- Thirty-five were able to RTS in the NHL (97%).
- The revision rate was 2.5%, and we have no information about graft types.

Sikka et al^20^ published on 47 NHL players.

- Five players did not return to play, and 4 were unable to return to play for a full season.
- Four players had a subsequent failure of reconstruction, and there was a total reoperation rate of 20% in this group.

There was no correlation between performance or complication rate and the type of graft used.

For baseball, there is a publication from Fabricant et al^9^ on 26 Major League Baseball position players.

- Twenty-three of 26 (88%) players were able to return to at least 30 games after ACL reconstruction.
- They had no information about graft types and revision rates.

Last but not least, we present some data on handball.

- Myklebust et al^18^ presented a cohort of 57 operatively treated Norwegian handball team players.
- Fifty-eight percent of them continued playing at the same level, 30% played at a lower level, and 7 players never played again.
- Of the 50 players who continued playing team handball, 11 reinjured their ACL, all when playing team handball.
- Forty-seven of the 57 players (82%) had a BTB graft reconstruction. In 8 cases, the ligament was sutured, and the procedure performed in 2 players was unknown.

## Discussion/Conclusion

The conclusions of our analyses of graft choice according to age and sport are the following:

- There is no perfect graft selection for everyone.
- When choosing a graft for ACL reconstruction, factors such as age, sex, sport type, and expectations should be taken into consideration and a personalized decision should be made.
- A review of the literature reveals a paucity of studies on the selection and outcomes of grafts in different sports.
- Given the unique characteristics of each sport, including the frequency of repetitive movements and specific performance expectations, it is clear that dedicated research is necessary for each discipline.
- Comparative studies on graft selection specific to each sport may increase the accuracy of the selection.

Thank you for your attention.
